# Galectin‐9 alleviates acute graft‐versus‐host disease after haplo‐hematopoietic stem cell transplantation by regulating regulatory T cell/effector T cell imbalance

**DOI:** 10.1002/iid3.1177

**Published:** 2024-02-14

**Authors:** Nannan Pang, Shabaaiti Tudahong, Yuejie Zhu, Jiang He, Chunxia Han, Gang Chen, Weiguo Wang, Jing Wang, Jianbing Ding

**Affiliations:** ^1^ Department of Pathology The First Affiliated Hospital of Shihezi University Shihezi China; ^2^ Center of Hematology, The First Affiliated Hospital of Xinjiang Medical University Xinjiang Uygur Autonomous Region Research Institute of Hematology Urumqi China; ^3^ Reproductive Fertility Assistance Center The First Affiliated Hospital of Xinjiang Medical University Urumqi China; ^4^ Department of Laboratory Medicine General Hospital of Xinjiang Military Region, PLA Urumqi China; ^5^ Department of Urology, Suzhou Hospital, Affiliated Hospital of Medical School Nanjing University Suzhou China; ^6^ Xinjiang Laboratory of Respiratory Disease Research Traditional Chinese Medicine Hospital Affiliated to Xinjiang Medical University Urumqi China

**Keywords:** acute graft‐versus‐host disease, haplo‐HSCT, galectin‐9/Tim‐3, PI3K/AKT/mTOR pathway, Treg/Th17 imbalance

## Abstract

**Background:**

Acute graft‐versus‐host disease (aGVHD) arises from the imbalance of host T cells. Galectin‐9 negatively regulates CD4 effector T cell (Th1 and Th17) function by binding to Tim‐3. However, the relationship between Galectin‐9/Tim‐3 and CD4^+^ T subsets in patients with aGVHD after Haplo‐HSCT (haploidentical peripheral blood hematopoietic stem cell transplantation) has not been fully elucidated. Here, we investigated the role of Galectin‐9 and CD4^+^T subsets in aGVHD after haplo‐HSCT.

**Methods:**

Forty‐two patients underwent Haplo‐HSCT (26 without aGVHD and 16 with aGVHD), and 20 healthy controls were included. The concentrations of Galectin‐9, interferon‐gamma (IFN‐γ), interleukin (IL)‐4, transforming growth factor (TGF)‐β, and IL‐17 in the serum and culture supernatant were measured using enzyme‐linked immunosorbent assay or cytometric bead array. The expression levels of Galectin‐9, PI3K, p‐PI3K, and p‐mTOR protein were detected by western blot analysis. Flow cytometry was used to analyze the proportions of CD4^+^ T cell subsets. Bioinformatics analysis was performed.

**Results:**

In patients with aGVHD, regulatory T (Treg) cells and Galectin‐9 decreased, and the Th1, Th17, and Treg cells were significantly imbalanced. Moreover, Treg and Galectin‐9 were rapidly reconstituted in the early stage of patients without aGVHD after Haplo‐HSCT, but Th17 cells were reconstituted slowly. Furthermore, Tim‐3 upregulation on Th17 and Th1 cells was associated with excessive activation of the PI3K/AKT pathway in patients with aGVHD. Specifically, in vitro treatment with Galectin‐9 reduced IFN‐γ and IL‐17 production while augmenting TGF‐β secretion. Bioinformatics analysis suggested the potential involvement of the PI3K/AKT/mTOR pathway in aGVHD. Mechanistically, exogenous Galectin‐9 was found to mitigate aGVHD by restoring the Treg/Teffs (effector T cells) balance and suppressing PI3K.

**Conclusion:**

Galectin‐9 may ameliorate aGVHD after haplo‐HSCT by modulating Treg/Teffs balance and regulating the PI3K/AKT/mTOR pathway. Targeting Galectin‐9 may hold potential value for the treatment of aGVHD.

## Graphical abstract

In patients with acute graft‐versus‐host disease (aGVHD), the expression of Tim‐3 is significantly increased. Galectin‐9 binding to Tim‐3 may inhibit the activation of the PI3K/AKT pathway and enhance the function of Treg cells. On the other hand, transforming growth factor (TGF)‐β promotes the differentiation of Treg cells through autocrine secretion, while TGF‐β induces the expression of Galectin‐9 in a paracrine manner. The increased Treg cells can inhibit the activation of Th1 and Th17 cells by secreting TGF‐β, thus alleviating aGVHD and inducing immune tolerance

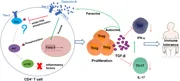

## 1 INTRODUCTION

Acute graft‐versus‐host disease (aGVHD) may occur in 30%–50% of patients who undergo transplantation from human leukocyte antigen (HLA)‐matched donors.^1^, ^2^, ^3^ The incidence of aGVHD is higher in patients with stem cell transplants from HLA‐mismatched and unrelated donors.^2^, ^3^ GVHD is characterized by the excessive activation of effector T cells (Teffs), including Th1, Th17, and cytotoxic CD8^+^T cells, which can result in the excessive secretion of cytokines, and ultimately induce inflammatory injury to target organs.^4^, ^5^

Galectin‐9 is a widely expressed soluble molecule that can bind to Tim‐3 and negatively regulate the immunity function of Teffs.^6^ By binding to Tim‐3 on Th1 and Th17 cells, it induces apoptosis and/or inhibits cell differentiation.^7^ The Tim‐3/Galectin‐9 pathway acts as a negative regulator of effector CD4^+^ and CD8^+^ T cells, thereby prolonging the lifespan of allogeneic skin grafts.^6^, ^8^, ^9^ Our previous study found that in patients with aGVHD who had received haploidentical peripheral blood hematopoietic stem cell transplantation (haplo‐HSCT), Tim‐3 expression was upregulated while serum Galectin‐9 was downregulated.^10^ However, the underlying mechanism of Tim‐3 and Galection‐9 in aGVHD remains unclear.

Shayan et al. found that the PI3K/AKT/mTOR pathway mediated cross‐talk between Tim‐3 and PD‐1.^11^ Tim‐3 can regulate PI3K‐AKT phosphorylation and activate downstream cell proliferation pathways.^12^, ^13^, ^14^, ^15^ The PI3K/AKT/mTOR pathway plays a crucial role in T cell activation and function. This pathway regulates many cellular events of T cells, including proliferation, survival, migration, and metabolism.^16^ Specifically, PI3K activation in T cells promotes cell survival and cell cycle progression, regulates differentiation, and controls the acquisition of effector and memory phenotypes.^17^, ^18^, ^19^, ^20^, ^21^ Overactivation of the PI3K/AKT pathway is often observed in organ transplantation, such as autologous orthotopic liver transplantation and kidney transplantation.^22^, ^23^, ^24^ The PI3K/AKT/mTOR pathway is considered a promising therapeutic target for preventing the development of GVHD.^25^, ^26^, ^27^ Herrero‐Sánchez et al.^27^ demonstrated that targeting the PI3K/AKT/mTOR pathway inhibited T cell activation and thus prevented GVHD development. Studies have shown that the rapid reconstitution of regulatory T (Treg) cells and the upregulation of TGF‐β and IL‐10 after haplo‐haplo‐HSCT can induce immune tolerance and decrease the risk of aGVHD.^28^, ^29^ The imbalance between Treg and Teffs is a major contributor to aGVHD.^30^, ^31^ The key to alleviating aGVHD is to restore the Treg/Teffs balance. To our knowledge, the relationship between PI3K/AKT/mTOR and Treg/Teffs balance in patients with aGVHD after haplo‐HSCT has not been reported. Therefore, we evaluated whether Galectin‐9 could modulate Treg/Teffs balance to ameliorate aGVHD after haplo‐HSCT. The PI3K/AKT/mTOR pathway was also analyzed and discussed. The findings suggest that targeting Galectin‐9 could potentially facilitate the treatment of aGVHD.

## 2 MATERIALS AND METHODS

### 2.1 Study participants

This study included 42 patients who underwent Haplo‐HSCT for hematological malignancies from December 2018 to August 2022. All transplantations were successful, with donor cell chimerism rates exceeding 95%. Hematopoietic reconstitution was achieved in all patients within 20 days posttransplantation, and blood samples were collected on Days 30, 45, 60, and 90 following transplantation. Peripheral blood was collected immediately upon occurrence of aGVHD before Day 30 post‐transplantation. Meanwhile, healthy controls (HC) (*n* = 20) were also enrolled, and their peripheral blood samples were also collected. We excluded patients who died or relapsed within 3 months after transplantation. Written informed consent was provided. This study was approved by the Ethical Committee of Xinjiang Medical University (20120220‐126).

### 2.2 Conditioning regimen and mobilization, collection, and transfusion of peripheral blood hematopoietic stem cells (PBSCs) from donors

The precondition and mobilization regimens were performed according to our previous studies.^28^, ^29^ The classic Ara‐c+Bu/Cy+ATG conditioning regimen and the Bu/Cy+ATG regimen were administered. Granulocyte colony‐stimulating factor (7–10 µg/kg.d) was used to mobilize peripheral PBSCs from donors, which were then collected on the 5th and 6th day post mobilization and infused to the recipients on the same day of collection. The infusion volume of mononuclear cells was (13.44 ± 5.23) × 10^8^/kg, and that of CD34+ cells was (7.53 ± 2.78) × 10^6^/kg.

### 2.3 aGVHD prophylaxis

An enhanced GVHD prophylaxis regimen was used, including CsA, Tac, MTX, anti‐CD25 mAb, MMF, DXMS, and MP, as previously described.^28^, ^29^ The aGVHD diagnosis and grading followed the Seattle criteria^32^ and the Chinese expert consensus on allogeneic hematopoietic stem cell transplantation for the treatment of hematological diseases (Ⅲ)—acute versus host disease.^33^ After confirmation of aGVHD, methylprednisolone (1 mg/kg) was used as the first line of treatment, with the dose adjusted based on the severity of aGVHD, and other immunosuppressants were used as necessary.

### 2.4 Flow cytometry

The proportions of CD4^+^ T subsets were analyzed with flow cytometry. CD4^+^ T subset biomarkers were selected as previously described.^34^ For the analysis of Th17, Th1, and Th2 cells, peripheral blood samples (100 μL) were incubated with CD4‐FITC, CCR6‐PerCP‐Cy^TM^5.5, CXCR3‐APC, and Tim‐3‐PE fluorescent‐labeled antibodies. For the analysis of Treg cells, the peripheral blood samples (100 μL) were incubated with CD4‐FITC, CD25‐APC, CD127‐PerCP‐Cy^TM^5.5, and Tim‐3‐PE fluorescent‐labeled antibodies. IgG‐PE was used as the isotype control. BD Biosciences provided the antibodies and reagents. The antibody incubation was performed at 4°C for 20 min in the dark, followed by red blood cell lysis for 5 min in the dark. After centrifugation and washing, the cells were resuspended and detected on a flow cytometer (Canto II, BD Biosciences). Fluorescence signals were excited at 488 nm and acquired using the 530/30‐A channel for FITC, 695/40‐A channel for PerCP‐Cy™5.5, and 575/25‐A channel for PE. For APC, fluorescence signals were excited at 633 nm and acquired using the 670/30‐A channel. At least 50,000 cells per sample were analyzed. Data analysis was conducted with the Flow Jo software (Tree Star) and Kaluza software (Beckman Coulter, Inc.). Lymphocytes were first gated using SSC and FSC. Then, CD4^+^ T cells were gated, followed by the analysis of each cell subset, including Th1 cells (CXCR3^+^CCR6^−^CD4^+^), Th2 cells (CXCR3^−^CCR6^−^CD4^+^), Th17 cells (CXCR3^−^CCR6^+^CD4^+^), and Treg cells (CD25^hi^CD127^low^CD4^+^).

### 2.5 Tim‐3^+^CD4^+^ T cell sorting

PBMCs were isolated from six patients with aGVHD using a lymphocyte separation solution (TBD Science). Subsequently, the Negative Selection CD4^+^ T‐cell sorting kit (STEMCELL Technologies) was used to enrich CD4^+^ T cells. The sorted CD4^+^T cells were re‐suspended to 5 × 10^7^/mL and incubated with Tim‐3‐PE antibody (BD, Clone:7D3, 2 µg/mL) in the dark for 15 min. Then, the incubation with 100 μL of EasySep® PE Selection Cocktail (STEMCELL Technologies) and 50 μL of EasySep® Magnetic Nanoparticles (STEMCELL Technologies) was performed. Finally, Tim‐3^+^CD4^+^ T cells were evaluated by flow cytometry, and their purity was over 90%.

### 2.6 Treatment of sorted Tim‐3^+^CD4^+^ T cells

The sorted Tim‐3^+^CD4^+^ T cells from patients with aGVHD were intervened with anti‐CD3 and anti‐CD28 (clone OKT3, clone CD28.2, eBioscience). After 48 h, the activated T cells were subjected to intervention with PBS, recombinant human Galectin‐9 (rhGalectin‐9) protein (ACRO Biosystems; 10 μg/mL), rhGalectin‐9 combined with goat anti‐human IgG (R&D Systems), rhGalectin‐9 combined with anti‐human Galectin‐9 antibody (clone 9M1‐3; Biolegend), or Rapamycin (100 ng/mL; Aladdin) for another 48 h. The cells and culture supernatants were subjected to Western blot and ELISA or cytometric bead array (CBA), respectively.

### 2.7 CBA

The serum was isolated from the peripheral blood samples. Interleukin (IL)‐17, interferon‐gamma (IFN‐γ), and IL‐4 levels in the serum and cell culture supernatant were determined using the human Th1/Th2/Th17 CBA Kits (BD). Finally, the samples were detected on a flow cytometer (Canto II, BD Biosciences). The data were analyzed using FCAP Array software version 3.0 (BD).

### 2.8 Enzyme‐linked immunosorbent assay (ELISA)

The levels of transforming growth factor (TGF‐β) and Galectin‐9 in the serum and culture supernatant were determined using ELISA kits (Thermo Fisher) (TGF‐β1: Cat# BMS249‐4; Galectin‐9: Cat# EH206RB), following the kit instructions. Blank control wells were set up. Finally, the absorbance of each well at 450 nm was measured on a microplate reader (Multiskan Go; Thermo Fisher). The levels of TGF‐β and Galectin‐9 were calculated according to the standard curves.

### 2.9 Western blot

PBMCs and sorted Tim‐3^+^CD4^+^ T cells were lyzed for protein extraction. The BCA method quantified protein concentration. Following separation, the proteins were transferred to the polyvinylidene difluoride (PVDF) membrane, which was then blocked for 1 h with 5% skimmed milk. Subsequently, the PVDF membrane was probed with primary antibodies overnight at 4°C. The anti‐human Vinculin primary antibody was from Proteintech Group (Cat# 26520‐1‐AP). The anti‐human Galectin‐9 was from Thermo Fisher (Cat# PA5‐50966). The β‐actin primary antibody was provided by Cell Signaling Technology. ABclonal Technology provided the PI3K p85α (cat#: A11526), Phospho‐PI3K P85α (Y467/Y199/Y464) (cat#: AP0854), and Phospho‐mTOR‐S2448 (cat#: AP0115) primary antibodies. Goat anti‐rabbit (Solarbio) horseradish peroxidase‐conjugated secondary antibody was incubated at room temperature for 1 h. The protein bands were developed using enhanced chemiluminescence (Biogot Technology). The gray values were analyzed by ImageJ software version 1.52 (NIH), and the relative protein expression was calculated.

### 2.10 Identification of differentially expressed genes

Peripheral blood samples were collected from patients with and without aGVHD (*n* = 3 each). PBMCs were isolated. The RNA was isolated from PBMCs and subjected to Illumina sequencing (Novogene). The sequencing results were analyzed using the DESeq. 2 software (1.20.0), and differentially expressed genes were screened using |log_2_^fold change [FC]^ | > 2 and *p* < .05.

### 2.11 Gene Ontology (GO) annotation, Kyoto Encyclopedia of Genes and Genomes (KEGG) pathway, and Gene Set Enrichment Analysis (GSEA)

Differentially expressed genes were analyzed for GO annotation and KEGG pathway enrichments using clusterProfiler (3.8.1). GSEA was performed online (https://maayanlab.cloud/Enrichr/; http://www.broadinstitute.org/gsea/index.jsp).

### 2.12 Statistical analysis

To describe the measurement data, we calculated the mean and standard deviation. We employed the Student's *t*‐test or analysis of variance followed by the LSD method, for the comparison of the data. Data with a heterogeneity of variance were analyzed with the rank sum test. The Spearman correlation test was used for correlation analysis. The data were processed with SPSS (version 22, SPSS Statistics/IBM Corp). *p* < .05 indicates a significant difference.

## 3 RESULTS

### 3.1 Baseline data

Table 1 shows the baseline data of the 42 patients with hematological malignancies. The recovery time of neutrophils ≥ 0.5 × 10^9^/L was (14.05 ± 3.88) days, and that of platelets ≥ 20 × 10^9^/L was (16.31 ± 6.92) days. Moreover, 26 cases did not have aGVHD, while 16 cases had aGVHD after transplantation, including 12 cases with mild aGVHD and four cases with severe aGVHD. The median time for aGVHD onset was 31 days after transplantation.

**Table 1:** Clinical characteristics of patients with related HLA haploidentical allogeneic peripheral blood hematopoietic stem cell transplantation (haplo‐HSCT).

Characteristics	Value
Number of cases	42
Age (years) (median, range)	30 (5.5–58)
Sex (%)	
Male/Female	25/17 (59.52/40.48)
Disease type (%)	
Acute lymphoblastic leukemia	9 (21.43)
Acute myeloid leukemia	22 (52.38)
Chronic myeloid leukemia	2 (4.76)
Myelodysplastic syndrome	1 (2.38)
Aplastic anemia	6 (14.29)
Hemophagocytic syndrome	2 (4.76)
Donor‐recipient relationship (%)	
Sibling	17 (40.48)
Parents for children	18 (42.86)
Children for parents	7 (16.66)
GVHD (%)	
aGVHD	16 (38.10)
Mild (I‐II)	12 (75.00)
Severe (III‐IV)	4 (25.00)
aGVHD onset time (days after haplo‐HSCT) (median, range)	31 (11–59)
Non‐aGVHD (%)	26 (61.90)
Infused graft	
MNC, ×10^8^/kg	13.44 ± 5.23
CD34+cells, ×10^6^/kg	7.53 ± 2.78

Abbreviations: aGVHD, acute graft‐versus‐host disease; haplo‐HSCT, haploidentical peripheral blood hematopoietic stem cell transplantation; HLA, human leukocyte antigen.

### 3.2 Immune reconstitution characteristics of CD4^+^T (Th1, Th2, Th17, and Treg) cell subsets after Haplo‐HSCT in patients without aGVHD

**Figure 1:**
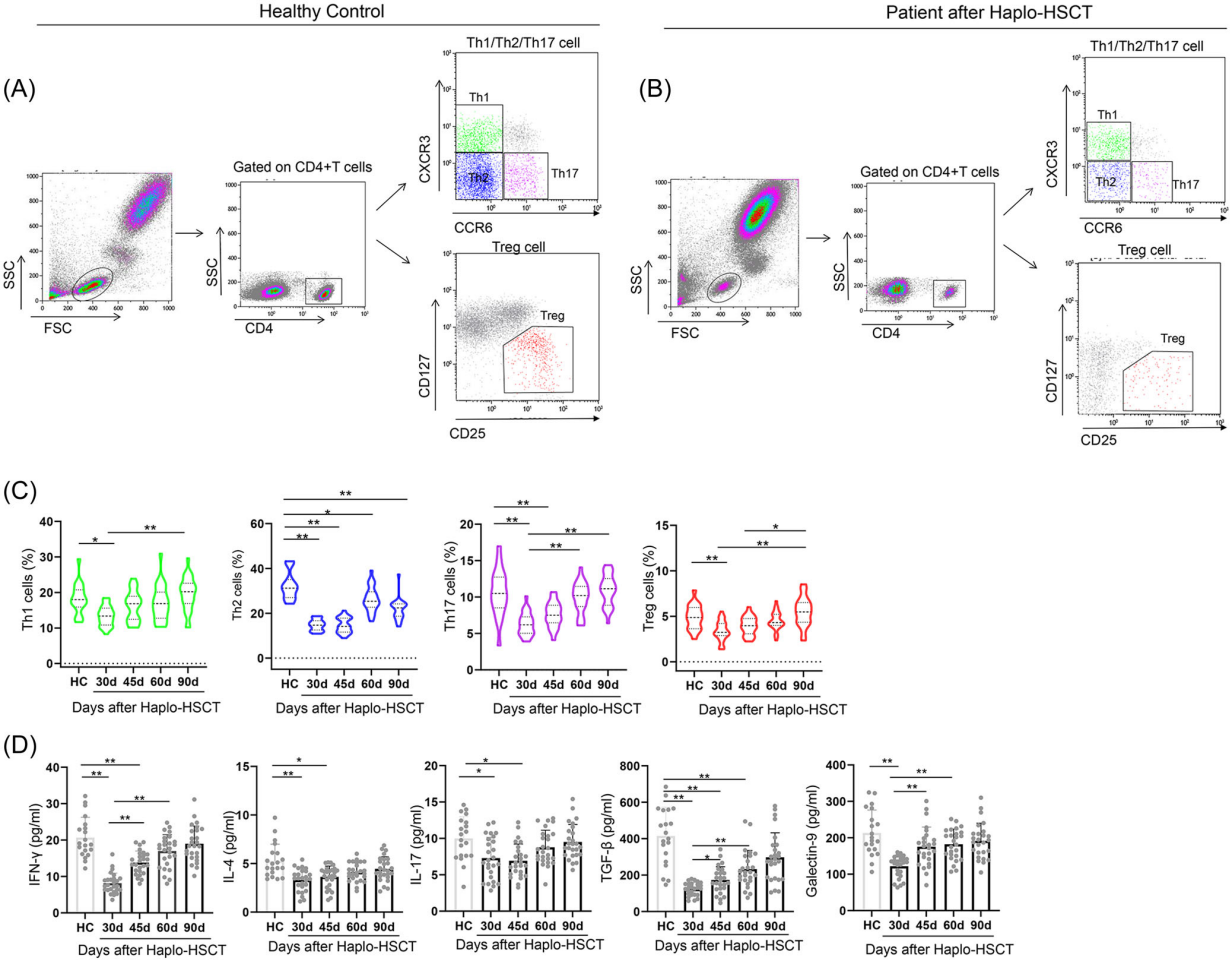
Dynamic alterations in peripheral blood CD4^+^T cell subsets in patients without aGVHD after transplantation. Percentage of Th1, Th2, Treg, and Th17 cells in patients without aGVHD. The percentages of Th1, Th2, Treg, and Th17 cells in total CD4^+^T cells were determined by flow cytometry. (A and B) Gating strategy. Treg cell was defined as CD4^+^CD25^hi^ CD127^low^. Th1 was defined as CD4^+^CXCR3^+^ CCR6^−^. Th2 was defined as CD4^+^CXCR3^−^CCR6^−^. Th17 was defined as CD4^+^ CXCR3^−^CCR6^+^. (C) Comparisons of the percentages of Th1, Th2, Treg, and Th17 cells in the peripheral blood of the HC, and patients after transplantation. The comparison was performed using a one‐way analysis of variance. Haplo‐HSCT, related HLA‐haploidentical peripheral blood hematopoietic stem cell transplantation; HC, healthy controls. *p < .05; ** *p* < .01. (D) Serum cytokine levels in patients without aGVHD after transplantation. Serum levels of IFN‐γ, IL‐4, TGF‐β, IL‐17, and Galectin‐9 in the patients from the Haplo‐HSCT group (on Days 30, 45, 60, and 90 after transplantation), as well as in the healthy control (HC) subjects, were detected with ELISA or CBA. *p < .05; ** *p* < .01. aGVHD, acute graft‐versus‐host disease; CBA, cytometric bead array; ELISA, enzyme‐linked immunosorbent assay; IFN‐γ, interferon‐gamma; IL, interleukin; TGF‐transforming growth factor.

Flow cytometry assessed the reconstitution of peripheral CD4^+^T cell subsets at 30, 45, 60, and 90 days after Haplo‐HSCT in patients without aGVHD. The gating strategy is shown in Figure 1A,B. Our previous study found that reconstitution of CD4^+^T cells began within 30 days after transplantation and gradually increased from 60 to 90 days posttransplantation.^28^ However, the CD4^+^T cell counts remained lower than those of healthy individuals. In this study, we found reconstitution of Treg and Th1 cells began 30 days after transplantation, and there was no significant difference at 60–90 days between each group and HC group (all *p* > .05, Figure 1C). Reconstitution of Th17 cells began 45 days after transplantation, and Th17 cells gradually increased and returned to normal levels 60 days after transplantation. On 30–90 days post‐transplantation, the number of Th2 cells was lower in comparison to the HC group (*p* < .05), indicating delayed immune reconstitution of Th2 cells. Hence, Treg and Th1 cells have faster immune reconstitution in the early posttransplantation period, Th17 cells begin to increase at 45 days after transplantation, and Th2 cells are relatively lagging in immune reconstitution.

### 3.3 The change of cytokines after transplantation in patients without aGVHD

CD4^+^T cell subsets exert biological effects by secreting different cytokines. In this study, the cytokines in the serum of patients without aGVHD were measured using CBA and ELISA. As shown in Figure 1D, compared with HC, the IL‐4, IFN‐γ, Galectin‐9, IL‐17, and TGF‐β in the serum of patients without aGVHD were markedly reduced on day 30 post‐transplantation. A gradually increasing trend in serum TGF‐β was observed on days 45 and 60 post‐transplantation. The level of Galectin‐9 in the serum increased on day 45, with no statistically significant difference compared to the control group (*p* > .05). Furthermore, the serum IL‐17, IFN‐γ, and IL‐4 were significantly lower than those in HC on days 30 and 45 post‐transplantation (*p* < .05). However, no significant differences between patients and HC were observed at other time points (*p* > .05). Therefore, the alterations of serum cytokines are in line with those of Th1, Th2, Treg, and Th17 cells. The serum level of Galectin‐9 has a slightly faster recovery than IL‐4, TGF‐β, IFN‐γ, and IL‐17.

### 3.4 Decreased Treg and imbalanced Treg/Th17 in peripheral blood of patients with aGVHD

**Figure 2:**
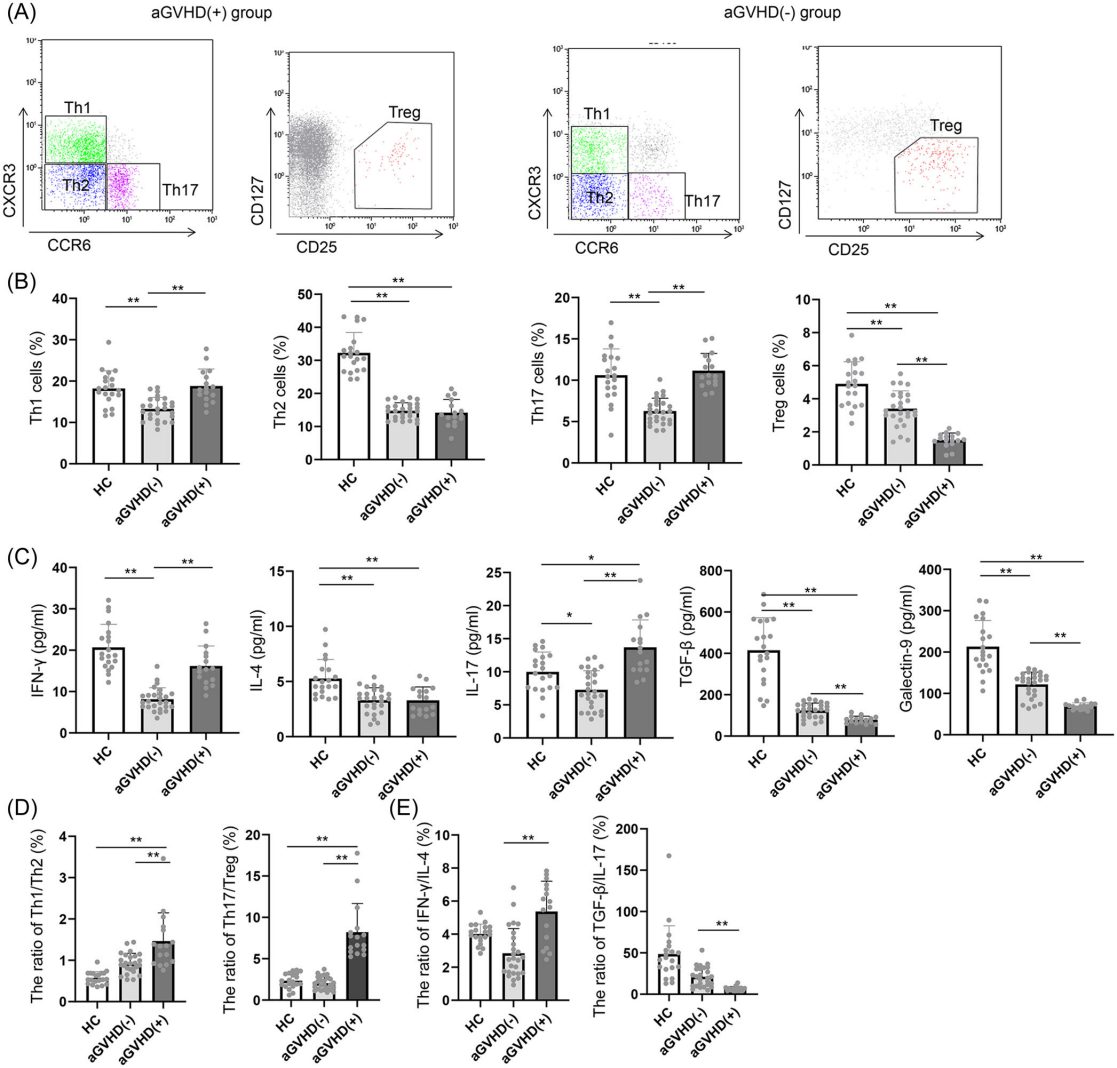
Percentage of Th1, Th2, Treg, and Th17 cells in the healthy control and patients with or without aGVHD. The percentages of Th cells in total CD4^+^T cell numbers were determined by flow cytometry. (A) Representative flow cytometry results. (B) Percentage of CD4^+^T cell subsets. (C) Cytokines changes in the healthy control and patients with or without aGVHD. Serum levels of Galectin‐9, IFN‐γ, IL‐4, TGF‐β, and IL‐17 were detected with ELISA or CBA. (D) Th1/Th2 and Treg/Th17 ratios. *p < .05; **p < .01. (E) Ratios of TGF‐β/IL‐17 and IFN‐γ/IL‐4. *p < .05, **p < .01. aGVHD, acute graft‐versus‐host disease; CBA, cytometric bead array; ELISA, enzyme‐linked immunosorbent assay; IFN‐γ, interferon‐gamma; IL, interleukin; TGF‐transforming growth factor.

Changes in CD4^+^T cell subsets were analyzed using flow cytometry in 26 patients without aGVHD (30 days post‐transplantation) and 16 patients with aGVHD (31 days posttransplantation). The results revealed that the Th1 and Th17 cell proportions were significantly higher in aGVHD patients (aGVHD(+)) than in patients without aGVHD (aGVHD(−)) (*p* < .01) (Figure 2A,B). Conversely, the aGVHD(−) patients had significantly increased Treg cell proportion than the aGVHD(+) patients (*p* < .01). The aGVHD(+) and aGVHD(−) patients (*p* > .05) did not differ significantly in Th2 cell proportion. The aGVHD(+) patients exhibited significantly elevated Th17/Treg and Th1/Th2 ratios than aGVHD(−) patients and HC (Figure 2D).

The analysis of cytokine levels revealed statistically elevated levels of IFN‐γ and IL‐17 in aGVHD(+) patients compared to aGVHD(−) patients (*p* < .01). Compared with the aGVHD(+) patients, the aGVHD(−) patients had significantly elevated Galectin‐9 and TGF‐β (*p* < .01, Figure 2C). IL‐4 level was similar between aGVHD(+) and aGVHD(−) patients. Notably, significant imbalances in both TGF‐β/IL‐17 and IFN‐γ/IL‐4 ratios were observed in aGVHD(+) and aGVHD(−) patients (Figure 2E). Thus, elevated levels of Galectin‐9 and TGF‐β were observed in aGVHD(−) patients, while aGVHD(+) patients showed elevated levels of IFN‐γ and IL‐17. These results indicate the imbalances of Treg/Th17 and Treg/Th1 cells in aGVHD patients.

### 3.5 Tim‐3 overexpression in patients with aGVHD

**Figure 3:**
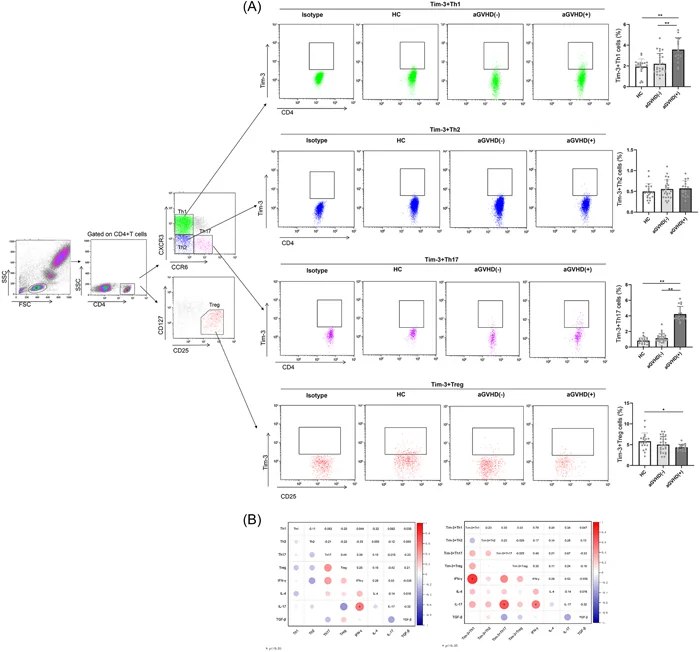
The expression of Tim‐3 on CD4^+^ T cell subsets. (A) Flow cytometry was performed to detect Tim‐3^+^Th1, Tim‐3^+^Th2, Tim‐3^+^Th17, and Tim‐3^+^Treg cells. Representative and quantitative flow cytometry results were shown. *p < .05, **p < .01. (B) Correlation analysis of cytokines and CD4^+^ T subsets in patients with aGVHD. Data were analyzed with Spearman correlation analysis. aGVHD, acute graft‐versus‐host disease.

Flow cytometry revealed that the aGVHD(+) patients had significantly elevated Tim‐3^+^Th1 and Tim‐3^+^Th17 proportions than the aGVHD(−) patients (Figure 3A) (*p* < .05). Nevertheless, Tim‐3^+^Treg and Tim‐3^+^Th2 cells were similar between groups (*p* > .05).

As shown in Figure 3B, the IFN‐γ and IL‐17 in patients with aGVHD were positively correlated with Tim‐3^+^Th1 and Tim‐3^+^Th17 cells, respectively (*r* = .78, *p* < .01; *r* = .67, *p* < .01). However, no significant correlation was found between other cytokines and other cell subsets (all *p* > .05). Hence, in patients with aGVHD, levels of IL‐17 and IFN‐γ in the peripheral blood may correlate with the numbers of Tim‐3^+^Th17/Th1 cells.

### 3.6 Genes associated with PI3K/AKT/mTOR signal pathway are upregulated after Haplo‐HSCT

**Figure 4:**
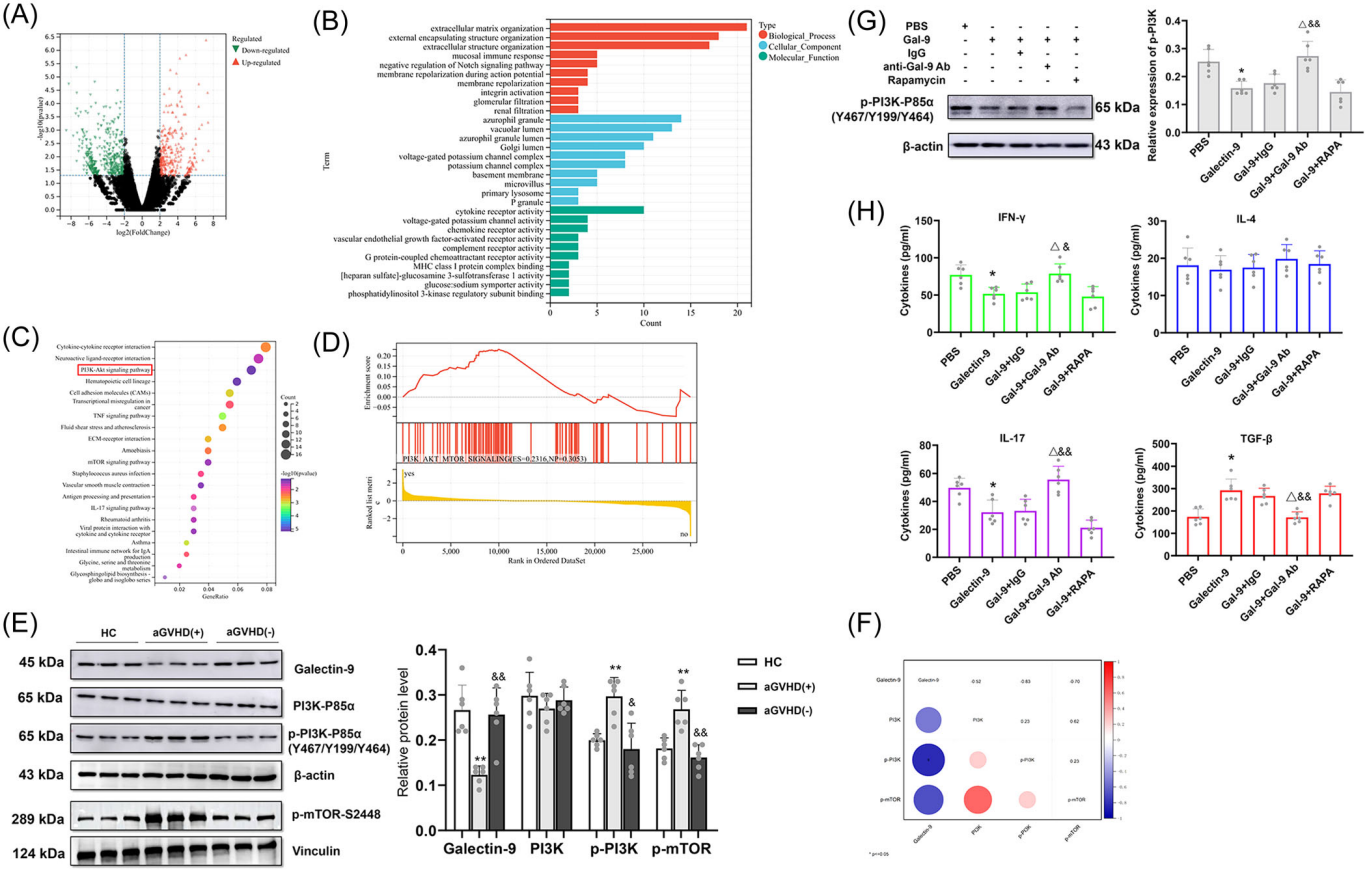
Gene expression profiles, functional analysis, and expression analysis of key proteins in PI3K/AKT/mTOR pathway in CD4^+^T cell subsets. (A) Volcano plot of differentially expressed genes between patients with or without aGVHD. (B) GO analysis of differentially expressed genes. (C) KEGG analysis of differentially expressed genes. (D) Gene Set Enrichment Analysis (GSEA) was used to analyze the signaling pathways (PI3K/AKT/mTOR signaling pathway) enrichment in different groups. (E) PI3K/AKT/mTOR pathway protein expression in the peripheral blood was measured by western blot. Compared with the control group, *p < .05, **p < .01; Compared with aGVHD(+) group, ^&^ *p* < .05, ^&&^ *p* < .01. (F) Correlation analysis in patients without aGVHD. Data were analyzed with Spearman correlation analysis. (G) Galectin‐9 downregulates p‐PI3K in the Tim‐3^+^CD4^+^T cells in vitro. Tim‐3^+^CD4^+^T cells from patients with aGVHD were treated with or without rhGalectin‐9 for 48 h. The levels of p‐PI3K were determined by western blot. Compared with the PBS group, *p < .05, **p < .01. Compared with the Galectin‐9 + IgG group, △*p* < .05, △△*p* < .01; Compared with Galectin‐9 + RAPA group, ^&^ *p* < .05, ^&^^&^ *p* < .01. (H) Analysis of IFN‐γ, IL‐4, IL‐17, and TGF‐β secretion by Tim‐3^+^CD4^+^T cells in vitro. Levels of IFN‐γ, IL‐4, IL‐17, and TGF‐β in culture supernatant were detected by ELISA. Compared with the PBS group, *p < .05, **p < .01. Compared with the Galectin‐9 + IgG group, △*p* < .05, △△*p* < .01; Compared with the Galectin‐9 + RAPA group, ^&^ *p* < .05, ^&^^&^ *p* < .01. aGVHD, acute graft‐versus‐host disease; CBA, cytometric bead array; ELISA, enzyme‐linked immunosorbent assay; GO, Gene Ontology; IFN‐γ, interferon‐gamma; IL, interleukin; KEGG, Kyoto Encyclopedia of Genes and Genomes; PBS, phosphate‐buffered saline; TGF‐transforming growth factor.

The aGVHD is associated with T‐cell activation and signaling pathway activation.^26^, ^27^ Here, we compared the transcriptomic profiles of patients with and without aGVHD (*n* = 3 each). The volcano plot showed 435 upregulated and 455 downregulated transcripts in patients with aGVHD (Figure 4A). GO analysis found that the genes with differential expression were mainly enriched in 115 GO terms of biological process (such as extracellular matrix organization) (*p* < .05), in 9 GO terms of cellular component (such as azurophil granule) (*p* < .05), and in 19 GO terms of molecular function (such as cytokine receptor activity) (*p* < .05) (Figure 4B). KEGG analysis showed that 21 pathways were significantly upregulated in aGVHD patients, including cytokine‐cytokine receptor interaction, IL‐17 signaling pathway, mTOR signaling pathway, PI3K‐AKT signaling pathway, and so forth (Figure 4C). Among them, the role of the PI3K/AKT/mTOR signaling pathway in aGVHD was significant (Figure 4D). Thus, it is necessary to further study the relationship between PI3K/AKT/mTOR and aGVHD.

### 3.7 PI3K/AKT/mTOR signaling pathway is possibly activated in aGVHD patients

As displayed in Figure 4E, p‐PI3K and p‐mTOR were upregulated significantly in the aGVHD(+) patients, in comparison to the aGVHD(−) patients and HC (*p* < .05). However, the aGVHD(−) patients exhibited significantly increased Galectin‐9 than the aGVHD(+) patients. PI3K was not significantly different. Moreover, correlation analysis revealed a significantly negative correlation between Galectin‐9 and p‐PI3K in the aGVHD(−) patients (*r* = −0.83, *p* <.05) (Figure 4F). Conversely, there was no significant correlation of Galectin‐9 with PI3K (*r* = −0.52, *p* = .288), and p‐mTOR (*r* = −0.7, *p* = .125). Therefore, the PI3K/AKT/mTOR signaling pathway is likely activated in patients with aGVHD, and high levels of Galectin‐9 may be associated with p‐PI3K.

### 3.8 Galectin‐9 inhibits PI3K/AKT by downregulating PI3K in the Tim‐3^+^CD4^+^T cells

We sorted out Tim‐3^+^CD4^+^T cells from aGVHD patients (*n* = 6) and cultured them in the presence of IgG, rhGalectin‐9, anti‐Galectin‐9 antibody, or Rapamycin for 48 h. Subsequently, we noted that p‐PI3K was reduced after treatment with Galectin‐9 or Galectin‐9+Rapamycin (*p* < .05) (Figure 4G). The group treated with anti‐Galectin‐9 antibody exhibited higher expression of p‐PI3K compared with the Galectin‐9+IgG group (*p* < .05). Thus, rhGalectin‐9 may suppress p‐PI3K in patients with aGVHD by inhibiting the PI3K/AKT signaling pathway.

### 3.9 The secretion of IFN‐γ and IL‐17 decreases significantly after the activation of the Tim‐3/Galectin‐9 pathway by rhGalectin‐9

ELISA and CBA revealed that the rhGalectin‐9 induced increased production of TGF‐β, but decreased secretion of IFN‐γ and IL‐17 by Tim‐3^+^CD4^+^T cells, compared with Galectin‐9 + anti‐Galectin‐9 and control (Figure 4H). Notably, this effect was reversed by the anti‐Galectin‐9 antibody. Conversely, the IL‐4 level was not significantly affected. Hence, the cytokine secretion of Tim‐3^+^CD4^+^ T cells is affected by rhGalectin‐9.

## 4 DISCUSSION

Both “cytokine storm” and excessive activation of T cells are considered the main contributors to aGVHD.^35^, ^36^ Previous studies have found that Th1 cytokine IFN‐γ in aGVHD patients is significantly upregulated and then decreased obviously after treatment, suggesting that serum IFN‐γ level is likely associated with aGVHD.^28^, ^29^ Tim‐3 on activated T cells could bind to Galectin‐9 and inhibit Teffs.^37^, ^38^ In this study, we found that there was an early and rapid immune reconstitution of Treg cells and Galectin‐9 levels in patients without aGVHD after Haplo‐HSCT, which may effectively prevent aGVHD. When aGVHD is present, the Galectin‐9/Tim‐3 negative pathway is not yet formed due to Galectin‐9 deficiency. Furthermore, in patients with aGVHD, there was high expression of Tim‐3 on effector CD4^+^T cells, and the production of cytokines (such as IL‐17 and IFN‐γ) was elevated after the activation of the PI3K/AKT/mTOR pathway. Finally, exogenous Galectin‐9 inhibited the activation of PI3K/AKT, thus alleviating aGVHD by reversing Treg/Teffs imbalance in vitro.

Restoration of innate and adaptive immunity is necessary for effective graft versus leukemia and for preventing post‐transplantation infections.^39^, ^40^ Ito et al. found that low‐dose Thymoglobulin suppressed the restoration of naïve T cells after allo‐HCT, but not the immune reconstitution of T cells.^41^ Here, our data demonstrated that hematopoietic reconstitution was achieved in all patients who received Haplo‐HSCT. Although ATG was used, Treg and Th1 cells in patients without aGVHD had a faster immune reconstitution in the early post‐transplantation period. Reconstruction of Treg can effectively prevent aGVHD. Recently, it has been revealed that early CD4^+^T cell immune reconstitution is related to patient survival with moderate to severe aGVHD.^42^ Cytokines secreted by different subsets of T cells after HSCT are involved in aGVHD.^43^ In this study, we demonstrated that patients who underwent Halpo‐PBSCT and were diagnosed with aGVHD exhibited an increase in Th1 and Th17 cells, but a reduction in Treg cells. Moreover, these patients had a significant decrease in the levels of Galectin‐9 and TGF‐β. This suggests that among aGVHD patients who underwent Halpo‐HSCT, there is an imbalance not only between Th1 and Th2 cells but also between Treg and Th17 cells, as well as an abnormal pattern of cytokine ratios IFN‐γ/IL‐4 and TGF‐β/IL‐17. In a mouse model of aGVHD, the overexpression of Tim‐3 was observed in CD4^+^ T and CD8^+^ T cells from the spleen and liver.^44^ Hansen et al found that Tim‐3 level was related to aGVHD severity.^45^ Here, our results demonstrated that there was Tim‐3 overexpression on Th1 and Th17 cells in patients with aGVHD after Haplo‐HSCT, suggesting that Th1 and Th17 cells may be abnormally activated in patients with aGVHD. Tim‐3 binding to Galectin‐9 can lead to intracellular calcium efflux and cell aggregation, which eventually results in the programmed cell death of Th17 and Th1 cells.^7^, ^37^ The interaction of Galectin‐9 and Tim‐3 can negatively regulate the response of Th1 and Th17 cells, and induce peripheral tolerance.^7^, ^46^ Nevertheless, in this study, Galectin‐9 was reduced significantly in patients who had aGVHD. We hypothesize that Galectin‐9 deficiency may lead to the absence of the Tim‐3/Galectin‐9 negative signaling pathway, thereby affecting Tim‐3's ability to exert a negative immunoregulatory function.

Studies have shown that Galectin‐9 can also interact with PD‐1 and VISTA.^47^, ^48^ Galectin‐9 facilitates T‐cell apoptosis through the cross‐linking of Tim‐3, while the co‐expression of PD‐1 attenuates Galectin‐9/Tim‐3‐induced apoptosis by promoting the formation of Tim‐3/Galectin‐9/PD‐1 lattices in cancer.^47^ Additionally, secreted Galectin‐9 alone can enhance PI3K activity in T cells, and downregulation results from the synergistic effect of Galectin‐9 with other immune checkpoint proteins such as VISTA (V‐domain immunoglobulin suppressor of T cell activation) and PD‐L1,^49^, ^50^ or small molecular weight compounds like l‐Kynurenine.^51^ PD‐1 can activate the protein phosphatase SHP‐2, which in turn downregulates the phosphorylation of crucial upstream kinases in T cells, resulting in decreased PI3K activity and thus regulating the PI3K/Akt/mTOR pathway.^52^ In this study, we found that 455 genes were downregulated and 435 genes were upregulated in patients with aGVHD. Additionally, the PI3K/AKT signal pathway was upregulated in patients with aGVHD. Through in vitro experiments, we revealed for the first time that in aGVHD patients, exogenous Galectin‐9 could bind to Tim‐3 on the cell surface and downregulate the phosphorylation level of PI3K in CD4^+^ T cells. This may affect the PI3K/Akt/mTOR pathway, thus inhibiting the function of effector CD4^+^ T cells. However, whether Galectin‐9, after haplo‐HSCT binds synergistically with PD‐1 and VISTA to downregulate the phosphorylation of upstream key kinases in T cells and inhibit PI3K activity is not yet clear, and further in‐depth research is warranted.

The Tim‐3/Galectin‐9 pathway plays a crucial role in promoting immunological tolerance in organ transplantation.^53^ In a study on mouse skin transplantation, exogenous Galectin‐9 protein downregulated the Tim‐3^+^Th1 immune response, alleviated inflammation, and ultimately prolonged the survival time of skin grafts.^8^ Galectin‐9 can inhibit T cell proliferation and specifically reduce IFN‐γ secretion in vitro.^7^ Another study found that Galectin‐9 (+) Th cells affected Th17/Treg cell balance by secreting Galectin‐9, similar to exogenous Galectin‐9.^54^ In our study, we found that exogenous rhGalectin‐9 significantly reduced the phosphorylation levels of PI3K and cytokine levels of IL‐17 and IFN‐γ. High doses of Galectin‐9 can inhibit Teffs, and thus a low serum level of Galectin‐9 poses a potential risk factor for aGVHD.^55^ In this study, activation of Tim‐3 with rhGalectin‐9 led to an enhanced function of Treg cells and caused a significant elevation of TGF‐β in vitro, which may reverse the Treg/Teffs imbalance.

Our study has some limitations. For example, the study had a small sample size. Moreover, the interaction of Galectin‐9 with PD‐1 and its synergistic effects with Tim‐3 was not studied through in vitro experiments. Further studies are warranted.

## 5 CONCLUSION

**Figure 5:**
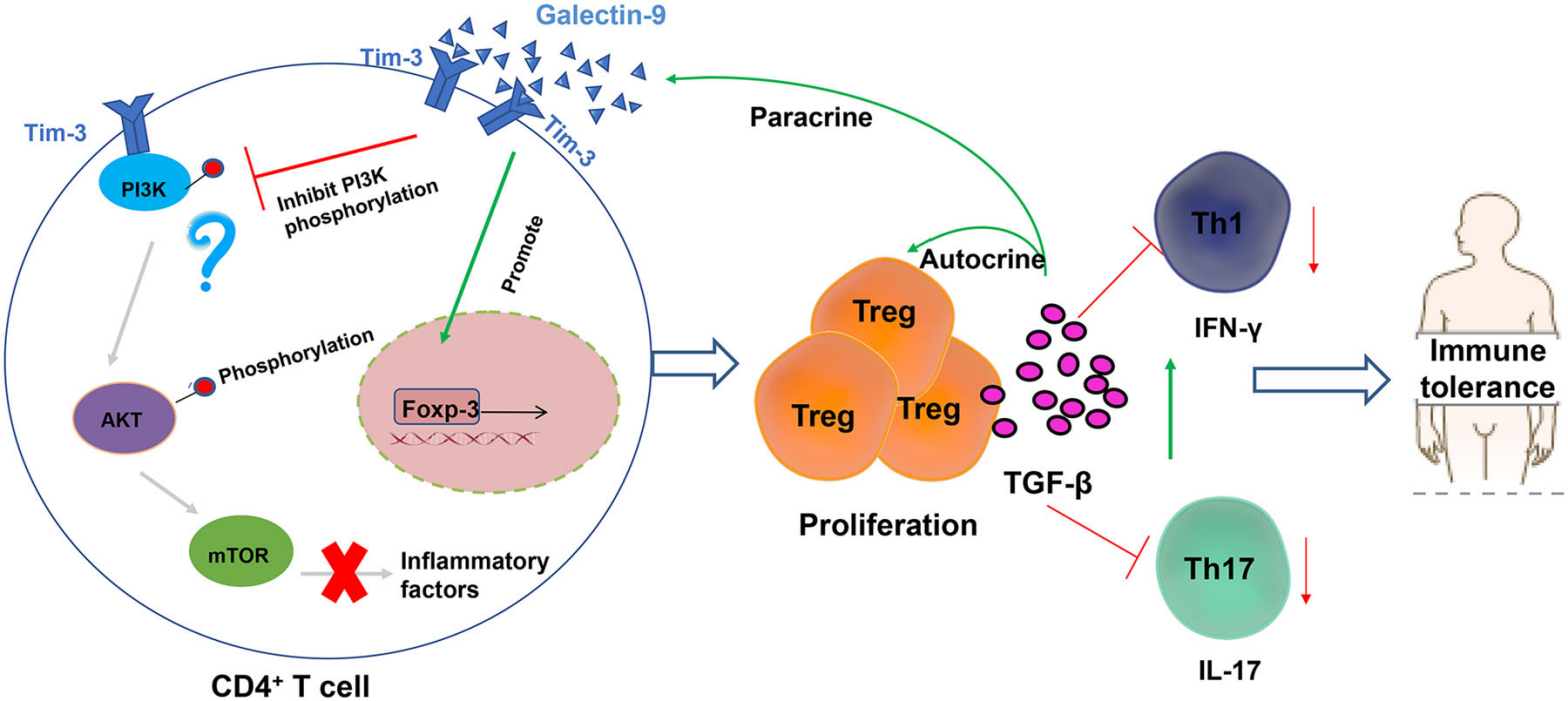
Schematic diagram illustrating the hypothesis of signaling regulation in aGVHD. In patients with aGVHD, the expression of Tim‐3 is significantly increased. Galectin‐9 binding to Tim‐3 may inhibit the activation of the PI3K/AKT pathway and enhance the function of Treg cells. On the other hand, TGF‐β promotes the differentiation of Treg cells through autocrine secretion, while TGF‐β induces the expression of Galectin‐9 in a paracrine manner. The increased Treg cells can inhibit the activation of Th1 and Th17 cells by secreting TGF‐β, thus alleviating aGVHD by inducing immune tolerance. aGVHD, acute graft‐versus‐host disease; IFN‐γ, interferon‐gamma; IL, interleukin; TGF‐transforming growth factor.

Following Haplo‐HSCT, the early immune reconstitution of Treg cells and the increased secretion of Galectin‐9 and TGF‐β can prevent the occurrence of aGVHD. Importantly, we have demonstrated that Galectin‐9 can inhibit the activation of CD4^+^ T cells in two ways. First, Galectin‐9 may inhibit the activation of the PI3K/AKT pathway and downregulate the expression of PI3K in Tim‐3^+^CD4^+^ T cells of aGVHD patients. Secondly, TGF‐β promotes the differentiation of Treg cells through autocrine secretion. On the other hand, TGF‐β induces the expression of Galectin‐9 in a paracrine manner, reversing the imbalance of Treg/Teff and thereby inducing immune tolerance and preventing the occurrence of aGVHD (Figure 5). Reconstitution of immune tolerance utilizing the Galectin‐9/Tim‐3 pathway may become a novel immune approach for the treatment and prevention of aGVHD in the future.

## AUTHOR CONTRIBUTIONS

Nannan Pang: Data curation; investigation (such as flow cytometry); formal analysis; writing—original draft; writing—review & editing. shabaaiti tudahong: data curation; investigation (such as western blot analysis); methodology. Yuejie Zhu: Data curation; investigation (such as cell sorting and in vitro experiments). Jiang He: Investigation (such as ELISA and CBA); resources. Chunxia Han: Investigation (such as collection of peripheral blood and serum specimens); formal analysis. Gang Chen: Investigation (such as collection of peripheral blood and serum specimens); formal analysis. Weiguo Wang: Supervision; project administration; funding acquisition; writing—review & editing. Jing Wang: Supervision; project administration; funding acquisition; writing—review & editing. Jianbing Ding: Conceptualization; supervision; project administration; writing—review & editing.

## CONFLICT OF INTEREST STATEMENT

The authors declare no conflict of interest.

## ETHICS STATEMENT

This study was approved by the Ethical Committee of Xinjiang Medical University (20120220‐126). Written informed consent was provided.

## Data Availability

The data that supporting the findings of this study are available upon reasonable request from the corresponding author.
